# Plasma Amylin and Cognition in Diabetes in the Absence and the Presence of Insulin Treatment

**DOI:** 10.4172/2155-6156.1000458

**Published:** 2014-10-23

**Authors:** Wei Qiao Qiu, Huajie Li, Haihao Zhu, Tammy Scott, Mkaya Mwamburi, Irwin Rosenberg, James Rosenzweig

**Affiliations:** 1 Department of Psychiatry, Boston University School of Medicine, USA; 2 Department of Pharmacology & Experimental Therapeutics, Boston University School of Medicine, USA; 3 Alzheimer’s Disease Center, Boston University School of Medicine, USA; 4 Department of Neurology, the First People’s Hospital of Chang Zhou, China; 5 Tufts University, USA; 6 Department of Medicine, Endocrinology, Boston University School of Medicine, USA

**Keywords:** Amylin, Cognition, Memory, Visuospatial and executive function

## Abstract

**Background:**

Plasma amylin is positively associated with cognitive function in humans. Amylin treatment improves memory in Alzheimer’s mouse models. However, the relationship between plasma amylin, diabetes and cognition is not clear.

**Objectives:**

In this study we examined the concentration of plasma amylin, its relationship with diabetes and cognition.

**Material and Method:**

A cross-sectional, homebound elderly population with data of plasma amylin under fasting condition and cognitive measurements was used.

**Results:**

We found that subjects with a long and chronic duration of diabetes were more likely to take insulin treatment and have reduced secretion of amylin. Compared to non-diabetics, diabetic subjects without insulin treatment had a higher concentration, but those with insulin treatment had a lower concentration, of plasma amylin [median (Q1, Q3): 20 (11.0, 36.2) vs. 25.2 (13.2, 50.6) vs. 15.0 (4.9, 33.8), p<0.0001]. In the whole sample vs. in the absence of diabetes, plasma amylin was positively associated with logical memory delayed recall (β= +0.61, SE=0.25, p=0.02 vs. β=+0.80, SE=0.33, p=0.02) and block design (β=+0.62, SE=0.24, p=0.009 vs. β=+0.93, SE=0.31, p=0.003), and negatively associated with Trailmaking A scores (β= −6.21, SE=1.55, p<0.0001 vs. β=−7.51, SE=1.95, p=0.0001) and Trailmaking B (β= −4.32, SE=2.13, p=0.04 vs. β= −5.86, SE=2.73, p=0.04). All these relationships disappeared in the presence of diabetes regardless the treatment.

**Conclusion:**

This study suggests that secretion of amylin by pancreas compensates and then deteriorates depending on the duration of diabetes. Amylin’s activities for cognition are impaired in the presence of diabetes.

## Introduction

Amylin is a gut-brain axis hormone with 37 amino acids produced and secreted by the pancreas. Amylin easily crosses the blood brain barrier (BBB) [1,2] and mediates important brain functions including inhibition of appetite through acting on area postremal [3], relaxation of cerebrovascular structure [4,5], and perhaps enhancement of neural regeneration [6]. It is co-secreted with another peptide, insulin, by the pancreas and plays an important role in regulating glucose metabolism [3]. Our recent study found a positive relationship between plasma amylin levels and the cognitive domains of memory and visuospatial function, suggesting a beneficial effect of this pancreatic peptide to brain function [7]. Patients with amnestic mild cognitive impairment (amnestic MCI) or Alzheimer’s disease (AD) have lower concentrations of plasma amylin than those with normal cognitive function [8].

Amylin shares several features with amyloid-beta peptide (Aβ), one hallmark component of AD pathology in the brain. Both peptides have similar secondary structure [9], both bind to the same amylin receptor [10], and both are degraded by the same protease, insulin degrading enzyme [IDE] [11,12]. Thus high levels of Aβ in the AD brain could compete with amylin to bind to its receptor interfering with amylin’s physiological activities in the brain. Using AD mouse models, preclinical studies including our own, Adler et al. [8] and Zhu et al. [13], found that peripheral injection of amylin or its clinical analog pramlintide improves learning and memory in these mice, suggesting an alternative therapeutic for AD.

Although all these studies suggest a protective effect of amylin for cognitive decline in the elderly, high concentration of plasma amylin is also associated with obesity and diabetes [7,14–16]. Obesity and diabetes are found to be associated with an increased risk of dementia including AD [17–22]. Thus it is critical to study the relationship between plasma amylin, diabetes and cognition in the elderly. We hypothesized that in type 2 diabetes, the dysfunctional pancreas compensates for amylin in a similar pattern it does for insulin by increasing its secretion at an early stage and declining greatly then diminishing leading to cognitive decline at a late stage of the disease. To prove that plasma amylin was associated with cognition depending on the disease duration and severity, we used a homebound elderly population and divided it into three subgroups: 1) those without diabetes, 2) diabetics without and 3) with insulin treatment. We then examined plasma amylin and cognition in these subgroups.

## Methods

### Study population and recruitment

We studied a group of 1062 subjects from a population-based study, the *Nutrition, Aging and Memory in the Elderly (NAME) study,* all of whom had been assessed for plasma amylin levels [23]. From this group, 190 subjects were excluded because they did not have plasma samples available for use (n=146) or did not have diabetes document (n=44) in this study. Those excluded from analysis had a similar rate of diabetes as the sample we used. Subjects included homebound elderly clients who were enrolled in one of four homecare agencies in the Boston area between 2002 and 2007. Anyone receiving homecare services was registered with one of these agencies if he/she lived in the city of Boston, had an annual income<$18,890, and needed homecare services. All homebound elders aged 60 and older at each of the four agencies were invited to participate in the study. All enrolled subjects gave informed consent. The protocol and consent form were approved by the Institutional Review Boards of Tufts University-New England Medical Center and Boston University School of Medicine.

### Clinical evaluation

Diabetes was defined as the use of anti-diabetic medication or fasting glucose greater than 126 mg/dl [19,24]. Subjects were asked to disclose all medications they were taking and the duration of the disease since the first time when they were informed of the diagnosis. Glucose concentrations were measured by the routine hexokinase method, and plasma insulin concentrations were measured by radioimmunoassay in the clinical laboratories.

Weight and height were measured twice using standardized instruments, and the average of two measurements was used to calculate BMI (kg/m^2^). Histories of stroke and cardiovascular diseases were self-reported.

### Measurements

#### Plasma amylin

Blood draw was conducted after 12 hours of fasting. Blood samples were centrifuged immediately to isolate plasma. We used an ELISA assay to measure amylin concentration in plasma according to the manufacturer’s instructions (Cat: EZHA-52K, LINCO Research, St. Charles, Missouri). All samples were assayed in duplicates and then averaged to give final values.

#### Other blood tests

Serum lipid profile including insulin, cholesterol, LDL and HDL, and serum creatinine were measured by the clinical laboratory according to the CDC standard protocols at Jean Mayer USDA Human Nutrition Research Center on Aging (HNRCA), Tufts University. Serum creatinine and C-reactive protein (CRP) levels were measured as a marker for renal function.

#### ApoE genotyping

A 244 bp fragment of the apoE gene including the two polymorphic sites was amplified by PCR with a robotic Thermal Cycler (ABI 877, Perkin-Elmer/Applied Biosystems), using oligonucleotide primers F4 (5′-ACAGAATTCGCCCCGGCCTGGTACAC-3′) and F6 (5′-TAAGCTTGGCACGGCTGTCCAAGGA-3′). The PCR products were digested with 5 units of Hha I and the fragments were separated by electrophoresis on 8% polyacrylamide non-denaturing gel. The specific allelic fragments were: E2; E3; and E4. ApoE4 was defined by E4/4, E3/4 or E2/4.

#### Cognitive function

Research assistants, trained by a board certified neuropsychologist, administered the cognitive tests. Cognition was assessed using a two-phase approach. 1) The population was screened for severe cognitive impairment using the Mini Mental State Examination (MMSE) [25] and for estimated verbal IQ or poor literacy using the North American Adult Reading Test (NAART) [26]. Those with MMSE<10 or verbal IQ<75 were not eligible to continue in the study. 2) Eligible subjects were subsequently examined using the following neuropsychological battery.

#### Verbal fluency (controlled oral word association test)

Total number of words generated beginning with a specific letter over 60 seconds, with three trials, each with a different letter. This test of phonemic generativity is usually viewed as a measure of executive functioning related to language ability (i.e., lexical access).

#### WAIS-III Digit Span Test

Both digits forward and digits backward were performed, and the total raw score was recorded. This test was used to evaluate attention/concentration (forward span), and working memory, which is another component of executive function (backward span).

#### WMS-III Word List Learning (WLL)

The task consisted of four learning trials of a 12 word list with an immediate recall score computed by summing the number of correct items recalled across all 4 trials. After a 30 minute delay, the subject was asked to recall the same list of words again, with the total correct items recorded to compute a delayed recall score. A percent retention score was calculated by dividing the number of words recalled on delay. These scores were used as measures of verbal learning and memory.

#### WMS-III Logical Memory (LM)

Two stories (A and B) were read aloud to the subject; the subject was then asked to repeat after each story, with all correctly recalled items totaled for an immediate recall score. After 30 minutes, the subject was asked to repeat both stories, total items correctly recalled comprised a delayed recall score. The ratio of the delayed recall score over the immediate recall score was used to calculate percent retention. These tests measured the different aspect of memory from WLL.

#### Trailmaking A

This test of visuomotor attention and processing speed requires participants to connect circles with numbers in them scattered across a page as quickly as possible. The time to completion is recorded, with a cap time of 301 seconds.

#### Trailmaking B

The subject was asked to draw a line connecting alternating letters and numbers in consecutive order. Time to completion was recorded, with cap time of 301 seconds. Trailmaking A time to completion was then subtracted from Trailmaking B total time to account for psychomotor speed and provide a more direct measure of executive function.

#### WAIS-III Block design

Subjects assembled red and white blocks to match a pictured design, with points assigned for each correctly replicated design and added together to compute a total score as a presumed measure of visuospatial skills.

### Statistical analysis

Statistical analysis was performed using SAS (version 9.3). Subjects were divided into three groups: a) those without diabetes, b) those with diabetes but not on insulin treatment, and c) those with diabetes and on insulin treatment. Continuous variables including plasma amylin concentration and cognitive analysis measures were characterized by diabetes status groups using ANOVA for normally distributed variables and Kruskal-Wallis Test for non-normally distributed variables to estimate the p values. The chi-squared test (and when required, Fisher’s exact test) was used to compare proportions for binary variables. For all analyses, the two-sided significance level of 0.05 was used.

Plasma amylin (Log Amylin) was transformed to log_10_ for multivariate regression due to skewed distribution. Linear regression was used to examine the associations between logged plasma amylin, diabetes status and cognitive function variables. Since there were few studies on the regulation of amylin in the literature, we assessed numerous factors that may affect amylin’s secretion and distribution on plasma amylin as possible confounding factors. Each set of variables was introduced manually and sequentially to reach the final model. Entry criteria into the model was p<0.2 and exit criteria was p>0.1. In addition, clinically significant variables were left in the model regardless of statistical non-significance. In the final model for the amylin’s relationship with diabetes status, adjustments were made for duration of the disease, insulin treatment, age, gender, ethnicity, education level, ApoE4 status, BMI, history of cardiovascular disease, history of stroke, kidney function (creatinine level), lipid profile including cholesterol, LDL and HDL levels, and other diabetic medications (Table 3). To study the relationship between plasma amylin and cognitive function as an outcome, we also used multivariate linear regression and adjusted for confounding factors including age, gender, ethnicity, education level, AD risk factor, and ApoE4 status (Tables 5 and 6). A 0.05 level of significance was used for all analyses.

## Results

### Study population

One thousand and sixty two subjects with documented diabetes status and measured levels of plasma amylin from the cross-sectional NAME study were used for this study analysis. The average age (mean ± SD) of this study sample was 75.0 ± 8.0 years old, and 76% were female. The population was multi-ethnic with 63% Caucasian, 35% African-American, and 2% other ethnicities. 66% had high school education or above and 23% carried at least one ApoE4 allele. We measured the concentrations of amylin and insulin in plasma: for amylin (pg/ml): median=21.5, Q1=11.0, Q3=39.4 and for insulin (pM/L), median=80.5, Q1=49.3, Q3=139.6.

### Characterization of diabetes and vascular complications in the NAME study

Table 1 shows that subjects were divided into three subgroups based on their diabetes status: 1) those who did not have diabetes, 2) those who had diabetes but had not been treated with insulin and 3) those who had diabetes and had been treated with insulin. The diabetic subgroup with insulin treatment were the youngest (mean ± SD: 71.2 ± 7.6 vs. 73.8 ± 8.0 vs. 76.1 ± 8.7, p<0.0001), had the most African-Americans (54% vs. 41% vs. 29%, p<0.0001), the lowest numbers who completed high school education (55% vs. 59% vs. 73%, p<0.0001), and the highest proportion with cardiovascular disease (58% vs. 50% vs. 36%, p<0.0001) and stroke (30% vs. 28% vs. 18%, p=0.02) compared to the diabetic subgroup without insulin treatment and the non-diabetic subgroup.

The diabetic subgroup with insulin treatment had the highest levels of serum glucose (mean ± SD: 142.4 ± 60.9 vs. 139.0 ± 46.7 vs. 97.7 ± 11.0, p<0.0001), creatinine (mean ± SD: 1.43 ± 1.19 vs. 1.06 ± 0.76 vs. 1.04 ± 1.03, p<0.0001) and C-reactive protein (CRP) [median (Q1, Q3): 8.0 (2.7, 14.8) vs. 3.8 (1.6, 8.9) vs. 3.0 (1.2, 8.0), p<0.0001], but the lowest levels of cholesterol (mean ± SD: 169.3 ± 50.2 vs. 178.4 ± 43.2 vs. 189.5 ± 41.3, p<0.0001), LDL (mean ± SD: 96.9 ± 40.2 vs. 101.6 ± 36.0 vs. 110.7 ± 34.6, p<0.0001) and HDL (mean ± SD: 42.5 ± 12.0 vs. 45.3 ± 12.4 vs. 52.5 ± 15.3, p<0.0001) compared to the diabetic subgroup without insulin treatment and the non-diabetic subgroup.

### Disease duration and plasma amylin

We then studied plasma amylin concentrations in three subgroups (Table 1). The diabetic subgroup without insulin treatment had higher level [median (Q1, Q3): 25.2 (13.2, 50.6)] of, but the diabetic subgroup with insulin treatment had lower level [median (Q1, Q3): 15.0 (4.9, 33.8)] of, plasma amylin, than the non-diabetic subgroup had [median (Q1, Q3): 20.0 (11.0, 36.2), p<0.0001]. The average of disease duration was longer in diabetes with insulin treatment than without insulin treatment (p<0.0001). We divided diabetic subjects into five subgroups based on the length of the disease (Table 2). Increasing duration of diabetes was positively associated with insulin treatment (p<0.0001) and negatively associated with amylin concentration (p<0.0002). While increasing length of disease was positively associated with the rate of cardiovascular disease, there were no differences in the rate of stroke and in the concentrations of glucose and insulin among different subgroups (data not shown). Using multivariate regression (Table 3), log_10_ of plasma amylin as an outcome remained to be negatively associated with duration of diabetes (β = −0.18, SE=0.06, p=0.002) and insulin treatment (β= −0.49, SE=0.19, p=0.03) after adjusting for diabetes, age, gender, ethnicity, education, ApoE4, BMI, cardiovascular disease, stroke, creatinine, the lipid profile including cholesterol, LDL and HDL and diabetic medications.

As expected, the diabetic subgroup with insulin treatment had the highest levels of plasma insulin [median (Q1, Q3): 174.9 (112.9, 240) vs. 94.9 (62.4, 166.3) vs. 69.3 (41.5, 110.6), p<0.0001] compared to the diabetic subgroup without insulin treatment and the non-diabetic subgroup as expected (Table 1). It was less likely for the diabetic elderly with insulin treatment to have been on metformin (22% vs. 39%, p=0.002) or sulfonylureas (10% vs. 52%, p<0.0001) than for those without insulin treatment.

### Diabetes, plasma amylin and cognitive function

Table 4 summarizes performances on the measures of cognitive functioning in each domain across the three subgroups and the comparisons between those diabetics without and with insulin treatment. While both diabetic subgroups had worse cognitive function in all the domains than those who did not have the disease, the diabetic subgroup with insulin treatment had the worst cognitive function. Those with insulin treatment had lower scores of MMSE (Mean ± SD: 24.2 ± 4.0 vs. 24.8 ± 3.6, p<0.0001), digit span (Mean ± SD: 12.7 ± 3.4 vs. 13.5 ± 3.5, p=0.03), block design (Mean ± SD: 17.9 ± 8.3 vs. 19.7 ± 9.3, p=0.003), and higher scores of Trailmaking A (Mean ± SD: Q1=94.4 ± 62.5 vs. 89.1 ± 61.6, p=0.05) and Trailmaking B (Mean ± SD: 232.4 ± 76.8 vs. 214.7 ± 84.5, p<0.0001) than those without the treatment. In contrast, there were no differences in the verbal fluency test and the memory performances between the two diabetic subgroups.

The effect of plasma amylin on cognition in diabetes could be attenuated in the presence of diabetes. To address this, we first studied the relationship between plasma amylin and cognition in the whole sample and then in the subjects who did not have diabetes (Table 5). Using multivariate regression, we found that log_10_ of plasma amylin was positively associated with some cognitive domains in the whole sample and more so among those who did not have diabetes, including LM delayed recall (whole sample: β=+0.61, SE=0.25, p=0.02 vs. no diabetic sample: β=+0.80, SE=0.33, p=0.02) and Block design (whole sample β = +0.62, SE=0.24, p=0.009 vs. no diabetic sample β=+0.90, SE=0.31, p=0.003), and negatively associated with Trailmaking A (whole sample: β= −6.21, SE=1.55, p<0.0001 vs. no diabetic sample: β= −7.51, SE=1.95, p=0.0001) and Trailmaking B (whole sample: β= −4.32, SE=2.13, p=0.04 vs. no diabetic sample: β= −5.68, SE=2.73, p=0.04), as an outcome after adjusting for age, gender, ethnicity, education and ApoE4. There were no associations between plasma amylin with MMSE and other cognitive tests including verbal fluency, digit span and word learning list. In contrast, among those who had diabetes regardless the treatment we did not find any association between plasma amylin and any cognitive tests (Table 6).

## Discussion

Our study suggests that low level of plasma amylin could be a mediating factor for cognitive decline during diabetes progression. Several large population-based studies have demonstrated that diabetes increases the risk of cognitive impairment [27–29], and the disease duration is associated with cognitive decline [17]. While the complication of cerebrovascular pathologies is thought to be the major etiology for cognitive decline in type 2 diabetes [30], not all diabetes elderly suffer from cognitive impairment compared to controls [31]. Our recent study demonstrates that soluble amylin from pancreas may be a protecting factor for cognitive decline in the elderly [7,13]. Amylin can aggregate and deposit in the pancreas in type 2 diabetes [32,33] that could lead to a low level of soluble amylin in plasma and impair amylin’s activities in the brain.

Compared to non-diabetics, diabetes with insulin treatment had a lower, but those without insulin treatment had a higher, average concentration of plasma amylin (Table 1). When type 2 diabetes patients suffer from the disease for a long duration, many of them need insulin treatment due to dysfunction of pancreas. As insulin and amylin are stored together and co-secreted by pancreas, amylin secretion would have a similar pattern as insulin by the damaged β cells in pancreas during chronic progression of type 2 diabetes, compensating by increasing its secretion and then diminishing its production [34]. This could explain the data shown in Tables 1, 2 and 3 that plasma amylin levels in diabetes were high when the average disease duration was shorter and the disease was milder so that insulin treatment was not needed; in contrast, the levels of plasma amylin were low regardless of insulin treatment when the diabetic duration was longer and the disease was severe.

The higher the concentrations of plasma amylin the elderly have, the better cognitive function they have, especially in the absence of diabetes (Table 5). This study showed that diabetic subjects with a long duration had poor cognition as well as a low concentration of amylin in plasma in the presence of insulin treatment (Tables 1 and 4). The rate of onset of AD is higher among patients who have suffered from type 2 diabetes for more than five years compared to those with a disease duration of less than five years [17]. Using Amyloid Precursor Protein (APP) transgenic mice, we observed that chronic intraperitoneal (i.p.) injection of AD animals with both amylin and its analog, pramlintide, reduce the amyloid burden as well as lowers the concentrations of Aβ and improve learning and memory [13]. Independently, Adler et al. demonstrate that a treatment with pramlintide improved performance in the novel object recognition task and increased expression of the synaptic marker synapsin I and the kinase cyclin-dependent kinase-5 in the hippocampus. In addition, amylin relaxes cerebral vasculature [4,5] and increases the blood supply to the brain [5].

Amylin readily crosses the BBB and mediates several activities including improving glucose metabolism, relaxing cerebrovascular structure, modulating inflammatory reaction and perhaps enhancing neural regeneration. Despite of these important functions, human amylin can form aggregates to disrupt islet structure in the pancreas in type 2 diabetes [32,35]. The relationship between plasma amylin and cognitive function disappeared among those with diabetes regardless insulin treatment (Tables 5 and 6). It is possible that amylin’s activities are impaired in the aggregating environment in type 2 diabetes even when the level of amylin is not low. One analog of amylin, pramlintide, has the substitution of prolines at positions 25, 28 and 29 of human amylin [36–39] so it has decreased potential for aggregation [40] and has become an antidiabetic drug. It has a favorable safety profile in clinical use, and only nausea is the most common tolerability-related adverse event [41]. Although pramlintide is an available drug for diabetes, at late stage of diabetes most diabetes patients have insulin treatment only without receiving pramlintide. One of probable reasons is that pramlintide treatment requires three subcutaneous injections in addition to insulin injections on daily basis leading to patients’ burden and discomfort. Our study suggests that amylin, natural or synthetic, may provide a new avenue for treatment of memory, psychomotor speed, visuospatial, and executive dysfunction in humans [7]. Currently there are no available treatments for memory, visuospatial and executive dysfunction in dementia, in diabetes, or in normal aging. Thus pramlintide treatment probably should be considered at least in diabetic patients who need insulin treatment and suffer from cognitive impairment.

The limitations of this study are: 1) this is a cross-sectional study, and we cannot conclude a protective relationship between a high concentration of amylin in plasma and attenuated cognitive decline; 2) this analysis does not include AD diagnosis, neuroimaging or autopsy measure, so the relationship between plasma amylin and brain structures or pathology is unknown; and 3) this study does not have hemoglobin A1C measurements which serve as a better biomarker to diagnose type 2 diabetes. Nevertheless, our results, in addition to those of other studies, suggest that a longitudinal study is needed to examine whether amylin is beneficial for preserving cognitive function in the elderly and for preventing development of AD.

## Figures and Tables

**Table 1:** Comparisons of demographic information, vascular diseases and lipid profiles across amylin quartiles.

	No diabetes	Diabetes, no insulin	Diabetes, + insulin	p values
	n = 674	n = 289	n = 99	
Age, year, mean ± SD	76.1 ± 8.7	73.8 ± 8.0	71.2 ± 7.6**	<0.0001
Female, n/total (%)	525/674 (78%)	208.289 (72%)	74/99 (75%)	N.S
African Americans, n/total (%)	195/671 (29%)	118/287 (41%)	53/99 (54%)	<0.0001
High School Graduate and above, n/total (%)	488/670 (73%)	170/287 (59%)	54/99 (55%)	<0.0001
ApoE4, n/total (%)	154/671 (23%)	63/286 (22%)	28/98 (29%)	N.S
BMI, Mean ± SD	29.8 ± 7.8	34.0 ± 8.8	37.1 ± 9.6**	<0.0001
Cardiovascular disease, n/total (%)	237/661 (36%)	140/281 (50%)	57/98 (58%)	<0.0001
Stroke, n/total (%)	122/663 (18%)	60/284 (28%)	30/99 (30%)	0.02
Glucose, mg/L, mean ± SD	97.7 ± 11.0	139.0 ± 46.7	142.4 ± 60.9	<0.0001
Creatinine, mg/dL, mean ± SD	1.04 ± 1.03	1.06 ± 0.76	1.43 ± 1.19***	<0.0001
Cholesterol, mg/dL, Mean ± SD	189.5 ± 41.3	178.4 ± 43.2	169.3 ± 50.2*	<0.0001
LDL, mg/dL, mean ± SD	110.7 ± 34.6	101.6 ± 36.0	96.9 ± 40.2	<0.0001
HDL, mg/dL, mean ± SD	52.5 ± 15.3	45.3 ± 12.4	42.5 ± 12.0*	<0.0001
CRP, mg/L, median (Q1, Q3)	3.0 (1.2, 8.0)	3.8 (1.6, 8.9)	8.0 (2.7, 14.8)**	<0.0001
Diabetes status				
Duration, n/total (%):	-			
<1 year		22/240 (9.2%)	0/99 (0%)	<0.0001
1–4 years		62/240 (25.8%)	6/99 (6.1%)	
5–9 years		62/240 (25.8%)	18/99 (18.2%)	
10–20 years		60/240 (25.0%)	38/99 (38.4%)	
>20 years		34/240 (14.2%)	37/99 (37.4%)	
Metformin, n/total (%)	-	113/289 (39%)	22/99 (22%)	0.002
Sulfonylureas, n/total (%)	-	151/289 (52%)	10/99 (10%)	<0.0001
Glitazones, n/total (%)	-	28/289 (10%)	8/99 (8%)	N.S
Meglit, n/total (%)	-	3/289 (1%)	1/99 (1%)	N.S
Peptides				
Insulin, pM/L, median (Q1, Q3)	69.3 (41.5, 110.6)	94.9 (62.4, 166.3)	174.9 (112.9, 240.0)***	<0.0001
Amylin, pM/dL, median (Q1, Q3)	20.0 (11.0, 36.2)	25.2 (13.2, 50.6)	15.0 (4.9, 33.8)***	<0.0001

Subjects are divided into three subgroups: those who did not have diabetes, diabetics without insulin and with insulin treatment. Mean ± SD with ANOVA test or n/total with Chi-Square test is used to describe the normal distributions and comparisons of age, diseases, kidney function assessed by the measurement of creatinine and the lipid biomarkers across three subgroups. P values for the statistical significance are described.

Mean ± SD with T-test is used to describe the distributions and comparisons of continuous variables between two diabetic subgroups. P values for the statistical significance are shown with * p<0.05; ** p<0.001; *** p<0.0001

BMI=Body Mass Index; LDL=low density lipoprotein; HDL=high density lipoprotein; CRP=C-reactive protein

**Table 2:** Comparisons of plasma amylin concentration and other variables among the diabetic subgroups with different disease duration.

Diabetes duration	<1 year	1–4 years	5–10 years	1–4 years	>20 years
	n=22	n=69	n=81	n=100	n=71
Amylin, pM/dL, median	26.2	28.2	26	17.9	13
(Q1, Q3)**	(13.4, 69.6)	(15.8, 57.3)	(11.4, 48.0)	(9.9, 41.4)	(5.8, 29.2)
Insulin treatment, n (%)***	0 (0.0)	6 (8.7)	18 (22.2)	38 (38.0)	37 (52.1)
Cardiovascular disease, n (%)*	13 (59.1)	24 (35.8)	42 (52.5)	56 (56.6)	44 (63.8)
Stroke, n (%)	3 (13.6)	14 (20.3)	19 (23.1)	31 (31.3)	20 (29.4)

Diabetic subjects are divided into three subgroups based on disease duration:<1 year; 1–4 years; 5–9 years; 10–20 years and >20 years. n/total with Fisher exact test or mean ± SD with ANOVA test or is used to describe the normal distributions and comparisons of those with insulin treatment, amylin concentration and the vascular complications across five subgroups.

P values for the statistical significance are shown with *p<0.05; **p<0.001; ***p<0.0001

**Table 3:** Multivariate regression analysis on the relationship between plasma amylin as an outcome and insulin use in diabetes.

	Log10 Amylin (n = 930)		Log10 Amylin (n = 930)	
	Model I		Model II	
	Estimate b (SE)	p value	Estimate b (SE)	p value
Diabetes	+0.43 (0.19)	0.02	+0.33 (0.24)	0.16
Diabetes duration	−0.17 (0.06)	0.002	−0.18 (0.06)	0.002
Insulin treatment	−0.53 (0.16)	0.0008	−0.49 (0.19)	0.03

Plasma amylin was transformed to log_10_ (Log_10_ Amylin).

Model I = adjusting for age, sex, ethnicity, education, ApoE4, BMI, cardiovascular disease, stroke, lengths of diabetes, creatinine (kidney function) and the lipid profile includes cholesterol, LDL and HDL.

Model II = Model I plus other diabetic drugs including metformin, sulfonylureas, glitazones and meglitinide

**Table 4:** Comparisons of cognitive function between non-diabetic and diabetic subgroups in the context of insulin treatment.

	No diabetes	Diabetes, no insulin	Diabetes, + insulin
	n=674	n=289	n=99
MMSE score, mean ± SD	25.5 ± 3.4	24.8 ± 3.6**	24.2 ± 4.0***
Verbal fluency, mean ± SD	28.6 ± 12.5	26.5 ± 12.3*	25.0 ± 11.8**
Digit span, mean ± SD	14.1 ± 3.8	13.5 ± 3.5*	12.7 ± 3.4***
WLL delayed recall, mean ± SD	3.7 ± 2.9	3.5 ± 2.8	3.4 ± 2.7
LM delayed recall, mean ± SD	19.0 ± 9.8	17.4 ± 9.8*	17.9 ± 9.4
Trailmaking A, mean ± SD	83.7 ± 57.4	89.1 ± 61.6	94.4 ± 62.5*
Trailmaking B, mean ± SD	205.3 ± 84.3	214.7 ± 84.5	232.4 ± 76.8***
Block design, mean ± SD	20.7 ± 9.1	19.7 ± 9.3	17.9 ± 8.3**

Subjects are divided into three subgroups: those who did not have diabetes, those who had diabetes without insulin treatment and those who had diabetes with insulin treatment. Mean ± SD with T-test is used to describe the distributions and comparisons of test scores in each cognitive domain between those who did not have diabetes and either diabetic subgroup. P values for the statistical significance are shown with *p<0.05; **p<0.01; ***p<0.001

WLL = Word learning list; LM = Logical memory

**Table 5:** Effects of plasma amylin on cognitive tests in multivariate regression analyses.

Log_10_	All Subjects		Subjects without diabetes	
Amylin				
Outcomes	Adjusting for age, gender, ethnicity, school and ApoE4		Adjusting for age, gender, ethnicity, school and ApoE4	
	Estimate β (SE)	P value	Estimate β (SE)	P value
MMSE Scores	+ 0.14 (0.09)	0.11	+ 0.16 (0.11)	0.15
Verbal fluency	+ 0.15 (0.33)	0.5	+ 0.54 (0.42)	0.21
Digit span	+ 0.05 (0.10)	0.6	+ 0.13 (0.13)	0.3
WLL delayed recall	+ 0.05 (0.07)	0.52	+ 0.04 (0.10)	0.71
LM delayed recall	+ 0.61 (0.25)	0.02	+ 0.80 (0.33)	0.02
Trailmaking A	− 6.21 (1.55)	< 0.0001	− 7.51 (1.95)	0.0001
Trailmaking B	− 4.32 (2.13)	0.04	− 5.68 (2.73)	0.04
Block design	+ 0.62 (0.24)	0.009	+ 0.93 (0.31)	0.003

Plasma amylin was transformed to log_10_ (Log_10_ Amylin) as a determining factor. MMSE = Mini Mental State Exam; WLL = Word learning list; LM = Logical memory

**Table 6:** Effects of plasma amylin on cognitive tests in multivariate regression analyses in the presence of diabetes.

	Diabetes		Diabetes with insulin treatment		Diabetes without insulin treatment	
Outcomes	Estimate β (SE)	P value	Estimate β (SE)	P value	Estimate β (SE)	P value
MMSE Scores	+ 0.13 (0.16)	0.39	+ 0.10 (0.24)	0.69	+ 0.10 (0.21)	0.65
Verbal fluency	− 0.02 (0.55)	0.97	− 0.60 (0.90)	0.51	+ 0.15 (0.74)	0.83
Digit span	− 0.10 (0.15)	0.53	+ 0.15 (0.24)	0.54	− 0.37 (0.20)	0.07
WLL delayed recall	+ 0.04 (0.12)	0.73	− 0.06 (0.19)	0.77	+ 0.08 (0.17)	0.65
LM delayed recall	+ 0.09 (0.43)	0.83	− 0.35 (0.65)	0.59	+ 0.52 (0.58)	0.37
Trailmaking A	− 4.92 (2.80)	0.08	− 6.62 (4.20)	0.12	− 3.81 (3.81)	0.32
Trailmaking B	− 2.23 (3.73)	0.55	− 4.63 (5.43)	0.4	+ 0.01 (5.02)	1
Block design	− 0.12 (0.40)	0.77	+ 0.09 (0.66)	0.89	− 0.21 (0.52)	0.69

Plasma amylin was transformed to log_10_ (Log_10_ Amylin) as a determining factor. These models were adjusted for age, gender, ethnicity, school year, ApoE4 and diabetes duration. MMSE = Mini Mental State Exam; WLL = Word learning list; LM = Logical memory
